# Die Mischung macht’s eben? Blended-Psychotherapie als Ansatz der Digitalisierung in der Psychotherapie

**DOI:** 10.1007/s00278-021-00524-3

**Published:** 2021-07-09

**Authors:** Laura Luisa Bielinski, Leonie Trimpop, Thomas Berger

**Affiliations:** 1 Abteilung Klinische Psychologie und Psychotherapie, Institut für Psychologie, Universität Bern, Fabrikstr. 8, 3012 Bern, Schweiz; 2 Lehrstuhl für Klinische Psychologie und Psychotherapie, Universität Witten/Herdecke, Witten, Deutschland

**Keywords:** Gemischte Therapie, Internetbasierte Intervention, Face-to-face-Psychotherapie, E‑Health, Psychische Gesundheit, Combined modality therapy, Internet-based intervention, Face to face psychotherapy, eHealth, Mental health

## Abstract

**Hintergrund:**

Über die letzten Jahrzehnte wurden verschiedene Ansätze zur Digitalisierung der Psychotherapie (PT) entwickelt. Eine Behandlungsform stellt die Kombination von „Face-to-face“-PT und Online-Interventionen, die „Blended-PT“, dar. Während das Forschungsinteresse zu Blended-PT in den letzten Jahren zugenommen hat, wurde die praktische Anwendung von Blended-PT im deutschsprachigen Raum bisher weniger stark umgesetzt. Auch bedingt durch die globale, durch die „coronavirus disease 2019“ (COVID-19) ausgelöste Pandemie gewinnen Blended-PT und andere Online-Ansätze zunehmend an Bedeutung.

**Ziel der Arbeit:**

Ein Überblick zum Thema und zu verschiedenen Formen von Blended-PT wird gegeben. Im Weiteren wird auf die Wirksamkeit, die Sicht der Patient:innen und Therapeut:innen sowie auf das Thema der Implementierung eingegangen.

**Material und Methoden:**

Narrative Übersicht der Literatur zum Thema Blended-PT; auf Basis einer umfassenden Suche werden wichtige Überlegungen und Befunde eingeordnet und beschrieben.

**Ergebnisse:**

Der Begriff der Blended-PT wird bisher uneinheitlich verwendet. In Anlehnung an Blended-Learning-Ansätze können „blends“ auf verschiedenen Ebenen stattfinden. Es kann zwischen ergänzenden und transformierenden Blends unterschieden werden. In transformierenden Blends verändert das Format die Face-to-face-PT grundlegend. Zu einigen Blended-PT-Formen gibt es bereits Wirksamkeitsbelege, zu anderen besteht dringender Forschungsbedarf. Im Vergleich zu Face-to-face-PT und reiner Online-Therapie könnte die Blended-PT verschiedene Vorteile bieten.

**Schlussfolgerung:**

Das Interesse an Blended-PT wächst aufseiten von Patient:innen und Therapeut:innen. Um evidenzbasierte Blended-PT erfolgreich anbieten zu können, bedarf es der engen Zusammenarbeit zwischen Wissenschaft, Institutionen, Therapeut:innen und Kostenträgern im Gesundheitssystem.

## Abstract (en)

**Background:**

Over the last decades various approaches to digitalization of psychotherapy (PT) have been developed. The concept of blended PT, the combination of face to face and online interventions is one such example. While research interest on blended PT has greatly increased in the past years, the implementation of blended PT has lagged behind in German speaking countries. Also, due to the global pandemic caused by the coronavirus disease 2019 (COVID-19), blended PT and other online approaches seem to be more relevant than ever before.

**Objective:**

The aim is to give an overview on the various forms of blended PT. Furthermore, the efficacy, the perspectives of patients and therapists as well as the topic of implementation are discussed.

**Material and methods:**

Narrative review of the literature on the topic of blended PT. Important considerations and findings are classified and described based on a comprehensive search.

**Results:**

Blended psychotherapy is a concept in need of definition. By taking inspiration from blended learning concepts, we propose that a definition of blended psychotherapy should consider different levels of blends and may also consider the transforming effect on face to face psychotherapy. In transforming blends face to face PT is fundamentally changed. For some forms of blended PT the effectiveness has already been confirmed but for others there is an urgent need for research. Compared to face to face PT and purely online therapy, blended psychotherapy could provide several advantages.

**Conclusion:**

The interest in blended PT is increasing among patients and therapists. To be able to successfully provide evidence-based blended PT, close cooperation between science, institutions, therapists and relevant stakeholders is needed.

In den letzten Jahrzehnten wurden zunehmend neue Technologien zur Behandlung von psychischen Erkrankungen entwickelt und angewandt. Online-Interventionen haben sich diesbezüglich in vielen Studien als wirksam erwiesen (Barak et al. 2008; Carlbring et al. 2018). Die „Blended-Psychotherapie“ (Blended-PT) bezeichnet die Kombination von „Face-to-face“-PT und Online-Interventionen. Dieser Beitrag setzt sich mit der Frage der Definition von Blended-PT auseinander und gibt einen Überblick zur Wirksamkeit, zur Sicht von Therapeut:innen und Patient:innen sowie zu ihrer Implementierung in die Praxis.

## Definitionen und Varianten

Der Begriff „blended“ wird im Bereich der Therapie psychischer Erkrankungen uneinheitlich verwendet. Zum Teil werden Bezeichnungen wie „blended psychotherapy“, „blended therapy“, „blended care“, „blended intervention“ oder auf Deutsch – „verzahnte PT“ oder „gemischte PT“ genutzt. Einige Autoren beschreiben mit dem Begriff „blended care“ die Integration von Offline- und Online-Elementen in einen Behandlungsprozess (Wentzel et al. 2016). Andere Autoren subsumieren unter „blended interventions“ auch „Stepped-care“-Ansätze oder die Online-Nachsorge, die auf eine Face-to-face-PT folgt (Erbe et al. 2017). Gemeinsam haben die verschiedenen Termini, dass sie eine Kombination von Face-to-face- und Online-Interventionen umfassen. Es stellt sich jedoch die Frage, wie diese Kombination genau realisiert wird.

### Merke.

Blended-PT meint die Kombination von Face-to-face-PT mit Online-Interventionen.

**Figure 1:**
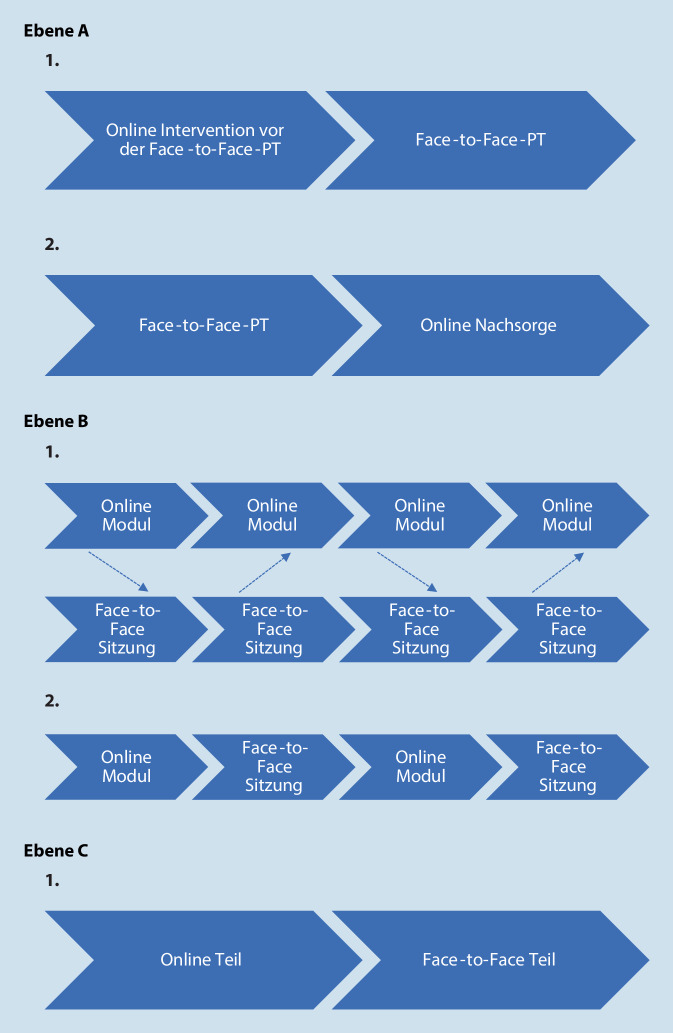

In Anlehnung an eine Einteilung in Ebenen aus dem Bereich des Blended Learning (Graham 2006) können auch „blends“ im Bereich der PT als Mischung von Face-to-face-PT und Online-Intervention auf verschiedenen Ebenen stattfinden. Es kann zwischen der Ebene der Gesamtbehandlung (beispielsweise Online-Interventionen der Face-to-face-PT vor oder nachgeschaltet), der Ebene der PT (beispielsweise Online-Intervention als Zusatz zur Face-to-face-PT oder Face-to-face-Sitzungen abwechselnd mit Online-Modulen) und der Ebene der Sitzung (beispielsweise Face-to-face- und Online-Teile in einer Sitzung) unterschieden werden (Abb. 1). Kombinationen der verschiedenen Ebenen sind ebenfalls möglich.

## Ein Beispiel einer Blended-Psychotherapie

**Figure 2:**
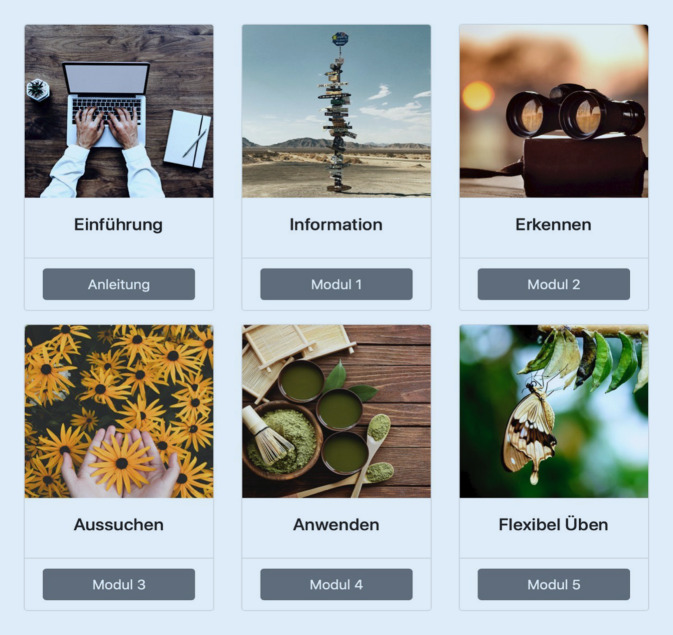

Eine laufende Studie der Autor:innen dieses Beitrags (Blended Transdiagnostic Intervention for Symptom Reduction and Improvement of Emotion Regulation in an Outpatient Psychotherapeutic Setting [REMOTION], Bielinski et al. 2020) untersucht mithilfe einer randomisierten kontrollierten Pilotstudie die „feasibility“ und erste Effekte einer Blended-PT auf der Ebene der PT (Abb. 1, *Ebene B, Punkt 1*). Die internetbasierte Intervention REMOTION wird als Zusatz zur Face-to-face-PT in der ambulanten Routinepraxis untersucht. Ziele der transdiagnostischen Online-Intervention sind es, die Emotionsregulation der Patient:innen zu verbessern und die Symptomreduktion zu fördern. Patient:innen erhalten dazu im Online-Teil umfassende Informationen zum Thema Emotionsregulation (Abb. 2). Der Online-Teil ist nach dem erweiterten Prozessmodell der Emotionsregulation nach Gross (2015) gegliedert. Die Patient:innen sollen zusätzlich zur Face-to-face-PT während insgesamt 6 Wochen ein Online-Modul/Woche bearbeiten. Therapeut:innen erhalten ebenfalls Informationen zu den einzelnen Modulen der internetbasierten Intervention (Tab. 1) und können ihre Elemente flexibel in die PT-Sitzungen integrieren.

**Table 1:** 

Komponenten		Beschreibung
Einführung		Patient:innen erhalten Informationen zum theoretischen Hintergrund der Intervention und eine technische Anleitung
Modul 1	Information	Patient:innen erhalten Informationen darüber, was Emotionen sind, welche Funktionen sie haben und welche Typen von emotionalem Erleben es gibt. Das Konzept der Emotionsregulation wird eingeführt
Modul 2	Erkennen	Dieses Modul verdeutlicht, wie Emotionen wahrgenommen werden können. Es werden verschiedene Übungen eingeführt. Zudem geht es um die Frage, ob und wann man Emotionen regulieren will
Modul 3	Aussuchen	Es werden Möglichkeiten zur Veränderung von über- und unterregulierten Zuständen (Bohus und Wolf-Arehult 2016; Greenberg 2015; Lynch 2018) sowie die Strategien der Situationsmodifikation, Aufmerksamkeitslenkung, kognitiven Bewertung und Reaktionsveränderung nach Gross (2015) eingeführt
Modul 4	Anwenden	Hier geht es um die Anwendung und das Üben der zuvor erlernten Strategien
Modul 5	Flexibel Üben	In diesem Modul geht es um das flexible und regelmäßige Anwenden von Emotionsregulationsstrategien

## Ergänzende vs. transformierende Blends?

**Figure 3:**
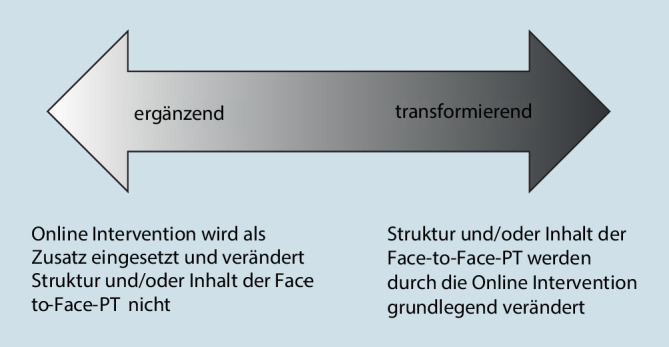

Interessanterweise wird im Bereich des Blended Learning neben der Unterscheidung von verschiedenen Ebenen auch der Begriff des „transforming blend“ (Graham 2006) eingeführt. Gemeint ist ein Blend, der die Art der Pädagogik radikal transformiert und damit intellektuelle Aktivität ermöglicht, die ohne ihn nicht stattgefunden hätte (Graham 2006). Im Bereich der Blended-PT kann zwischen transformierenden und ergänzenden Blends unterschieden werden (Abb. 3).

Beispielhaft handelt es sich um einen transformierenden Blend, wenn die Online-Intervention den Fokus und die Arbeit in den Face-to-face-Sitzungen verändert, indem das Online-Programm den Inhalt und die Struktur der Face-to-face-Sitzungen mitbestimmt oder bestimmte Themen nur noch online behandelt und andere online bereits eingeführte Themen in den Face-to-face-Sitzungen besonders vertieft werden. Insgesamt kann davon ausgegangen werden, dass eine stärkere Verzahnung der Face-to-face- und Online-Elemente zu einer stärkeren Transformation der Therapie führt. Im Gegensatz dazu werden die Online-Programme in ergänzenden Blends als Zusatz eingesetzt, wobei die Struktur und der Inhalt der Face-to-face-PT wenig tangiert werden sowie die Online- und Face-to-face-Elemente insgesamt meist wenig verzahnt sind.

### Merke.

Im Bereich der Blended-PT kann neben der Differenzierung von verschiedenen Ebenen auch zwischen ergänzenden und transformierenden Blends unterschieden werden.

Typischerweise ist der Grad der Transformierung auf der Ebene der Gesamtbehandlung am niedrigsten und auf der Ebene der Sitzung am höchsten, wobei es diesbezüglich sicherlich auch Ausnahmen gibt. Beispielsweise kann eine vorgeschaltete Online-Intervention im Rahmen eines Stepped-care-Ansatzes die darauffolgende PT auch verändern (spezifische Inhalte werden in der Face-to-face-PT nicht mehr thematisiert oder besonders in den Fokus gerückt etc.). Im Rahmen der Studie zu REMOTION (Bielinski et al. 2020) wird mithilfe der qualitativen Inhaltsanalyse von Interviewdaten u. a. die ergänzende vs. transformierende Wirkung der Blended-PT aus zwei unterschiedlichen Perspektiven, der Perspektive der Patient:innen und der Therapeut:innen, auch explorativ untersucht.

## Wirksamkeitsstudien

In den letzten Jahren wurden verschiedene Studien zur Wirksamkeit von Blended-PT publiziert (Berger et al. 2018; Nordgreen et al. 2016; Wright et al. 2005). Die meisten dieser Studien basieren auf der kognitiven Verhaltenstherapie (KVT) und wurden bei Depression, Angststörungen und Substanzabhängigkeit erforscht. Im Folgenden werden Beispiele für Studien zu Blends innerhalb der Gesamtbehandlung, der PT und auf der Ebene von Sitzungen dargestellt.

### Blends in den einzelnen Therapieebenen

#### Gesamtbehandlung

Mehrere Studien haben Online-Interventionen im Rahmen von Stepped-care Ansätzen (gestufte Therapie) oder als Nachsorge untersucht. Eine Studie von Nordgreen et al. (2016) untersuchte beispielsweise die Wirksamkeit eines Stepped-care-Modells bei Patient:innen mit sozialer Angst- oder Panikstörung. Die Patient:innen in der Stepped-care-Bedingung starteten mit einer Face-to-face-Psychoedukationssitzung; bei fehlendem Ansprechen konnten sie an einer geleiteten internetbasierten Intervention, basierend auf Prinzipien der KVT, teilnehmen. Danach folgte – falls die Online-Intervention ebenfalls nicht zum Erfolg führte – eine Face-to-face-KVT. Von den 34 Patient:innen, bei denen die Stepped-care-Bedingung Erfolg zeigte, sprachen 26 bereits nach den beiden weniger intensiven Behandlungsstufen ausreichend auf die Therapie an. Insgesamt profitierten die Patient:innen, die in die Stepped-care-Bedingung randomisiert wurden, gleich stark wie die Patient:innen in der Kontrollbedingung mit direkter Face-to-face-KVT. Die Abbruchraten waren im Stepped-care-Modell jedoch höher als in der reinen Face-to-face-Bedingung (41,2 % im Vergleich zu 27,3 %).

Fuhr et al. (2018) untersuchten die Wirkung einer internetbasierten Intervention während der Wartezeit auf einen ambulanten Therapieplatz. Die Interventionsgruppe erhielt, im Vergleich zur Kontrollgruppe, während der Wartezeit Zugang zu einem evidenzbasierten Online-Programm mit der Möglichkeit, während der Intervention automatisierte SMS-Nachrichten zu erhalten. Die depressive Symptomatik sank in beiden Studiengruppen während der Wartezeit, es gab jedoch keine signifikante Symptomverbesserung. Das Interesse an der Studienteilnahme war klein (nur die Hälfte der kontaktieren Personen war an einer Teilnahme interessiert), und die Personen, die das internetbasierte Programm nutzten, durchliefen im Schnitt nur die Hälfte aller Module.

Ein systematisches Review von Hennemann et al. (2018) untersuchte die Wirksamkeit von internet- und mobilbasierten Ansätzen als Nachsorge und Rückfallprävention. Insgesamt wurden 16 Studien zu Depression, Essstörungen und transdiagnostischen Ansätzen eingeschlossen, wobei nicht alle dieser Studien eine Nachsorge oder Rückfallpräventionen untersuchten, die direkt auf eine psychotherapeutische Behandlung folgte. Die Effektstärken bewegten sich zwischen klein und mittelgroß (Cohens *d* = −0,08 bis −0,45) bezüglich der Symptomausprägung nach den Interventionen im Vergleich zu den Kontrollgruppen. Insgesamt diskutierten die Autoren die suboptimale methodologische Qualität der Studien als Problem (Hennemann et al. 2018).

#### Psychotherapie

Eine Studie von Berger et al. (2018) konnte zeigen, dass ein Blend auf der Ebene der PT wirksamer ist als PT allein. Die Autor:innen untersuchten den Einfluss eines Online-Selbsthilfeprogramms als Zusatz zur PT in der ambulanten Routinepraxis auf die Symptomatik depressiver Patient:innen. Nach 12 Wochen Behandlung war die Blended-PT bezüglich der Veränderung depressiver Symptome wirksamer (Cohen’s *d* = 0,51) als PT allein. Die Autoren führen dies auch darauf zurück, dass die Patient:innen in der Blended-PT-Bedingung durch den Online-Zusatz nachweislich eine höhere Behandlungsdosis erhielten.

Eine andere Studie konnte zeigen, dass die Wirksamkeit einer Blended-PT auch bei stationären Patient:innen gegeben war (Zwerenz et al. 2017). Patient:innen erhielten zusätzlich zu einer psychodynamisch-orientierten, stationären Therapie entweder Zugang zu einem Online-Programm (Interventionsgruppe) oder Zugang zu reiner Online-Psychoedukation. Nach 12 Wochen gab es einen signifikanten Unterschied die depressive Symptomatik betreffend zwischen den beiden Gruppen, mit einem Vorteil für die Interventionsgruppe (moderater Effekt, *d* = 0,44). Darüber hinaus wurde die Überlegenheit der Blended-PT in Bezug auf die Angstsymptomatik, Lebensqualität und auf den Selbstwert der Patient:innen deutlich. Positive Effekte der Blended-PT fanden sich auch noch 6 Monate nach Studienbeginn (Zwerenz et al. 2019).

#### Sitzung

In der Studie von Wright et al. (2005) war eine Blended-PT-Bedingung, im Vergleich zu einer Warteliste, zur Behandlung der depressiven Symptomatik gleich wirksam wie eine Face-to-face-PT. In dieser Studie wurde in der Interventionsgruppe ein Blend auf der Ebene der Sitzung angewandt (Abb. 1, *Ebene C*). Patient:innen sahen während der Sitzungen zuerst ihre Therapeut:innen (erste Sitzung 50 min, danach immer 25 min) und bearbeiteten direkt anschließend während ca. 20–30 min ein internetbasiertes Programm. Nach 8 Wochen war die Behandlung abgeschlossen. Die Blended-PT beanspruchte weniger Therapeut:innenzeit als die Face-to-face-PT. Ähnliche Ergebnisse konnten in einer größeren Studie erhoben werden, die die Blended-PT mit Face-to-face-PT verglich, wobei dort die Blended-PT-Bedingung 16 Wochen dauerte und der Blend in den letzten 8 Wochen nicht strikt auf die Ebene der Sitzung begrenzt war, da Patient:innen das internetbasierte Programm nach Belieben nutzen konnten (Thase et al. 2018). Bezüglich Blends auf der Sitzungsebene gibt es bisher deutlich weniger Forschung als in den anderen Bereichen.

#### Fazit

Zusammenfassend liegen Hinweise auf die Wirksamkeit von Blended-PT auf den Ebenen der Gesamtbehandlung, der PT und der Sitzung vor. Auf der Ebene der Gesamtbehandlung scheinen jedoch wie bei reinen Online-Interventionen die Abbruchraten (Nordgreen et al. 2016) sowie die mangelnde Nutzung der Online-Elemente problematisch zu sein (Fuhr et al. 2018). Hier wäre also zu überlegen, wie die Online-Elemente im Sinne eines transformierenden Blend besser in die Gesamtbehandlung integriert und damit auch begleitet werden können. Beispielsweise könnte ein Online-Programm zur Nachsorge nach einem stationären Aufenthalt bereits während des Klinikaufenthalts eingeführt und in die bestehenden therapeutischen Konzepte integriert werden, und nach dem Austritt könnten die Möglichkeit bestehen, Therapeut:innen weiter online zu kontaktieren.

### Akzeptanz durch Patient:innen und Therapeut:innen

Für die Implementierung von Blended-PT ist nicht nur die Wirkung, sondern auch die Akzeptanz durch die Betroffenen wichtig. Verschiedene qualitative Studien haben die Einstellungen von Patient:innen gegenüber Blended-PT untersucht. Urech et al. (2019) führten Interviews mit 15 an Depressionen erkrankten Patient:innen durch, die ein Online-Selbsthilfeprogramm als Zusatz zur Face-to-face-KVT nutzten. Sie konnten zeigen, dass die Patient:innen Blended-PT als wirksam einschätzten, die internetbasierten und die PT-Elemente sich gut ergänzten und die Behandlung als effizient wahrgenommen wurde. Als mögliche Nachteile wurden eine fehlende Motivation für die Nutzung der Online-Elemente sowie zu wenig Integration der Online- mit den PT-Elementen identifiziert. Die Autoren ergänzten, dass Therapeut:innen möglicherweise bei gewissen Patienten*innen eine aktivere Rolle im Verzahnungsprozess einnehmen müssten, damit dieser erfolgreich sein kann. Dieses Defizit war z. T. der hohen Arbeitslast der Therapeut:innen und institutionellen Bedingungen geschuldet.

Ähnliche Resultate fand eine Studie von Etzelmüller et al. (2018); die Autor:innen führten Interviews mit Teilnehmenden einer Blended-PT, die sowohl internetbasierte Elemente als auch videobasierte PT beinhaltete, durch. Die Patient:innen schätzten u. a. die Möglichkeit, von zu Hause an der Behandlung teilnehmen zu können, sowie die Begleitung durch Therapeut:innen. Als negativ wurden u. a. technische Probleme sowie die empfundene emotionale Distanz in den Videosprechstunden bewertet.

Therapeut:innen sind gegenüber Blended-PT positiver eingestellt als gegenüber vollständig via Internet durchgeführten Therapien (Schuster et al. 2020). Sie sehen bei der Blended-PT weniger Risiken und Einschränkungen als bei der rein internetbasierten Therapie (Schuster et al. 2020). Ebenfalls gibt es diese Einstellungen interessante, länderspezifische Unterschiede betreffend. Therapeut:innen in Schweden sind z. B. gegenüber internetbasierter und Blended-PT positiver eingestellt als Therapeut:innen in Deutschland (Schuster et al. 2020). Dies könnte u. a. mit dem Grad der Unterstützung solcher Formate durch die jeweiligen länderspezifischen Gesundheitssysteme zusammenhängen (Schuster et al. 2020). Folgende Faktoren wurden in einer Studie zu Therapeut:innenperspektiven als förderlich für die Implementierung von Blended-PT beschrieben (Titzler et al. 2018):

- Blended-PT soll individuell auf Patient:innen zugeschnitten werden können;
- Das Verhältnis und die Anzahl von Online- und PT-Sitzungen sollen von Therapeut:innen ausgewählt werden können;
- Schulung/Training für Therapeut:innen;
- Konzepte zur Integration von Blended-PT im Gesundheitssystem sowie finanzielle Unterstützung;
- hochentwickelte technische Lösungen.

## Mögliche Vorteile gegenüber Face-to-face- und reiner Online-Psychotherapie

Im Vergleich zur Face-to-face-PT sowie zu reiner Online-Therapie könnte eine Blended-PT i. Allg. verschiedene Vorteile haben (Tab. 2). Es erscheint jedoch wichtig zu präzisieren, welche Ebene von Blended-PT genau gemeint ist. Während die genannten Vorteile auf der Sitzungsebene und der Ebene der PT klar ersichtlich sind, sind diese auf der Ebene der Gesamtbehandlung z. T. nicht anwendbar, weil die Online-Intervention zeitlich versetzt zur Face-to-face-PT und, wie schon erwähnt, oft ohne gleichzeitige Unterstützung durch Therapeut:innen genutzt wird.

**Table 2:** 

Im Vergleich zur Face-to-face-PT	Im Vergleich zur Online-Therapie
*Abwechslungsreiches Therapieangebot* Den Patient:innen kann ein breiteres Therapieangebot gemacht werden	*Face-to-face-Kontakt mit Therapeut:innen* Patient:innen können Face-to-face-Sitzungen in Anspruch nehmen
*Mehr Zeit für Vertiefung in den Face-to-face Sitzungen* Blended-PT erlaubt die Auslagerung von gewissen Elementen in die Online-Komponenten der Therapie	*Geeigneter in Krisen* Wegen des Face-to-face-Kontakts können Blended-Formate auch bei schwerer Belastung und in Krisensituationen genutzt werden
*Flexible Arbeit (unabhängig von Ort und Zeit)* Patient:innen können unabhängig von Ort und Zeit mit den Online-Komponenten der Therapie arbeiten	*Bessere Adhärenz und weniger Therapieabbrüche* Schwierigkeiten von reinen Online-Therapien bestehen in der teils geringe Adhärenz und hohen Abbruchraten
*Weniger Therapeut:innenzeit nötig* Falls Komponenten der Sitzungen oder einzelne Sitzungen durch Online-Elemente ersetzt werden	*Einstellung der Therapeut:innen* Therapeut:innen sind Blended-PT gegenüber positiver eingestellt als gegenüber Online-Therapie

## Offene Fragen und zukünftige Entwicklungen

Welche Kombination von Face-to-face-PT und Online-Intervention ist hilfreich? Welches Medium soll für welche Interventionen genutzt werden? Diese und ähnliche Fragen sollten in Zukunft vermehrt mit theoriegeleiteten Studien erforscht werden. Ein möglicher Ansatzpunkt ist der Forschungsbereich der computervermittelten Kommunikation. So nimmt beispielsweise die Theorie der medialen Reichhaltigkeit (Reichwald 1998 in Berger 2015) an, dass die Kommunikation bei einer guten Passung zwischen Aufgabenkomplexität und Reichhaltigkeit des Mediums am effizientesten ist. Komplexe Aufgaben benötigen reichhaltigere Medien, einfachere Aufgaben weniger reichhaltige (Berger 2015). Eine Blended-PT könnte also beispielsweise psychoedukative Elemente (weniger komplexe Aufgabe) internetbasiert gestalten und problemaktualisierende Elemente (komplexere Aufgabe) face to face bearbeiten. Ähnliche Überlegungen (praktische Elemente wie Psychoedukation eher in den Online-Modulen und prozessbezogene Elemente eher face to face) stammen von Van der Vaart et al. (2014).

Ebenfalls würden sich transdiagnostische und störungsspezifische Elemente der Behandlung mithilfe der Blended-PT neu kombinieren lassen. Dies könnte beispielsweise als Therapiemethode für Patient:innen mit Komorbiditäten vorteilhaft sein. Die Vorteile von transdiagnostischen Therapien, z. B. die Erreichung einer größeren Patientenpopulation (Schaeuffele et al. 2021), könnten ebenfalls mit den Vorteilen von Blended-PT (Tab. 1) kombiniert werden.

Neben der Frage nach der Wirksamkeit stellt sich die Frage der Indikation. Bei welchen Patient:innen und unter welchen Umständen ist Blended-PT indiziert? Bereits vor einigen Jahren haben sich Wentzel et al. (2016) mit dieser Frage beschäftigt. Dazu haben sie die Checkliste „Fit for Blended Care“ erstellt. Diese kann vor dem Einsatz von Blended-PT konsultiert werden. Therapeut:innen können sich so Gedanken über die individuelle Passung der Therapieform machen.

Inwiefern wird die Psychotherapie durch den Blended-Ansatz transformiert? Der Grad der Transformierung der Face-to-face-PT durch Blended-PT sollte in zukünftigen Studien explizit erhoben und berichtet werden. Die Entwicklung von geeigneten Erfassungsmethoden ist diesbezüglich nützlich. Interessant ist außerdem der Wirksamkeitsvergleich zwischen ergänzenden und transformierenden Blends.

Wie kann die Implementierung von Blended-PT in der Praxis unterstützt werden? In Anbetracht der aktuellen pandemiebedingten Einschränkungen ist mit einem Zuwachs von Online- und Blended-PT zu rechnen. Um einen „Wilden Westen von eHealth“ (Ruwaard und Kok 2015), wie in den Niederlanden beschrieben, zu vermeiden, ist jedoch eine verantwortungsbewusste, evidenzbasierte Implementierung von Blended-PT zentral.

### Merke.

Um die Qualität von Blended-PT zu sichern, ist eine verantwortungsbewusste, evidenzbasierte Implementierung von Blended-PT zentral.

Vonseiten der Wissenschaft sind Studien, die nicht nur die Wirksamkeit untersuchen, sondern auf Implementationsfragen fokussieren, essenziell. Für Psychotherapeut:innen ist eine Sensibilisierung für die Grenzen und Möglichkeiten von Blended-PT im Rahmen der Weiterbildung und Fortbildung unabdingbar. Vonseiten der Kostenträger im Gesundheitssystem ist die finanzielle Unterstützung von evidenzbasierter Blended-PT zentral.

## Fazit für die Praxis

- Blended-Psychotherapie (Blended-PT) gewinnt zunehmend an Wichtigkeit. Erste Wirksamkeitsbelege für verschiedene Ebenen von Blended-PT liegen vor, es gibt jedoch weiteren Bedarf an Replikationen und Studien, beispielsweise zu transformierenden Blends.
- Bei Blends auf Ebene der Gesamtbehandlung ist es wegen der Abbruchraten und der fehlenden Nutzung der Online-Angebote zentral, dass diese aus einem Guss entstehen: die Online-Interventionen gut in die Gesamtbehandlung integriert und von Therapeut:innen begleitet werden.
- Patient:innen interessieren sich für Blended-PT. Therapeut:innen scheinen gegenüber Blended-PT positiver eingestellt zu sein als gegenüber reiner Online-Therapie.
- Blended-Psychotherapie könnte im Vergleich zu reiner Online-Therapie und im Vergleich zur Face-to-face-PT verschiedene Vorteile haben, die Ebene des Blend muss jedoch mitberücksichtigt werden.
- Unterstützend für die erfolgreiche Implementierung sind das breitflächige Zur-Verfügung-Stellen von evidenzbasierten Online-Angeboten für Therapeut:innen, die Sensibilisierung und Unterstützung von Therapeut:innen betreffend Integration von Online-Angeboten in die Face-to-face-PT sowie die Unterstützung von Blended-PT durch die Kostenträger im Gesundheitssystem.

## References

[CR1] Barak A, Hen L, Boniel-Nissim M, Shapira NA (2008). A comprehensive review and a meta-analysis of the effectiveness of internet-based psychotherapeutic interventions. J Technol Hum Serv.

[CR2] Berger T (2015). Internetbasierte interventionen bei psychischen störungen.

[CR3] Berger T, Krieger T, Sude K, Meyer B, Maercker A (2018). Evaluating an e-mental health program (“deprexis”) as adjunctive treatment tool in psychotherapy for depression: results of a pragmatic randomized controlled trial. J Affect Disord.

[CR4] Bielinski LL, Krieger T, Moggi F, Trimpop L, Willutzki U, Nissen C, Berger T (2020). REMOTION blended transdiagnostic intervention for symptom reduction and improvement of emotion regulation in an outpatient psychotherapeutic setting: protocol for a pilot randomized controlled trial. JMIR Res Protoc.

[CR5] Bohus M, Wolf-Arehult M (2016). Interaktives Skillstraining für Borderline-Patienten.

[CR6] Carlbring P, Andersson G, Cuijpers P (2018). Internet-based vs. face-to-face cognitive behavior therapy for psychiatric and somatic disorders: an updated systematic review and meta-analysis. Cogn Behav Ther.

[CR8] Erbe D, Eichert HC, Riper H, Ebert DD (2017). Blending face-to-face and internet-based interventions for the treatment of mental disorders in adults: systematic review. J Med Internet Res.

[CR9] Etzelmueller A, Radkovsky A, Hannig W, Berking M, Ebert DD (2018). Patient’s experience with blended video- and internet based cognitive behavioural therapy service in routine care. Internet Interv.

[CR10] Fuhr K, Fahse B, Hautzinger M, Gulewitsch MD (2018). Erste Erfahrungen zur Implementierbarkeit einer internetbasierten Selbsthilfe zur Überbrückung der Wartezeit auf eine ambulante Psychotherapie [Implementation of an internet-based self-help for patients waiting for outpatient psychotherapy—First results]. Psychother Psychosom Med Psychol.

[CR11] Graham CR, Bonk CJ, Graham CR (2006). Blended learning systems. The handbook of blended learning: Global perspectives, local designs.

[CR12] Greenberg LS (2015). Emotion-focused therapy: coaching clients to work through their feelings.

[CR13] Gross JJ (2015). Emotion regulation: current status and future prospects. Psychol Inq.

[CR14] Hennemann S, Farnsteiner S, Sander L (2018). Internet- and mobile-based aftercare and relapse prevention in mental disorders: a systematic review and recommendations for future research. Internet Interv.

[CR15] Lynch TR (2018). Radically open dialectical behavior therapy: theory and practice for treating disorders of overcontrol.

[CR16] Nordgreen T, Haug T, Öst LG, Andersson G, Carlbring P, Kvale G, Tangen T, Heiervang E, Havik OE (2016). Stepped care versus direct face-to-face cognitive behavior therapy for social anxiety disorder and panic disorder: a randomized effectiveness trial. Behav Ther.

[CR18] Ruwaard J, Kok R (2015). Wild West eHealth: time to hold our horses?. Eur Health Psychol.

[CR19] Schaeuffele C, Schulz A, Knaevelsrud C, Boettcher J (2021). CBT at the crossroads: the rise of transdiagnostic treatments. J Cogn Ther.

[CR20] Schuster R, Topooco N, Keller A, Radvogin E, Laireiter AR (2020). Advantages and disadvantages of online and blended therapy: replication and extension of findings on psychotherapists’ appraisals. Internet Interv.

[CR21] Thase ME, Wright JH, Eells TD, Barrett MS, Wisniewski SR, Balasubramani GK, McCrone P, Brown GK (2018). Improving the efficiency of psychotherapy for depression: computer-assisted versus standard CBT. Am J Psychiatry.

[CR22] Titzler I, Saruhanjan K, Berking M, Riper H, Ebert DD (2018). Barriers and facilitators for the implementation of blended psychotherapy for depression: a qualitative pilot study of therapists’ perspective. Internet Interv.

[CR23] Urech A, Krieger T, Möseneder L, Biaggi A, Vincent A, Poppe C, Meyer B, Riper H, Berger T (2019). A patient post hoc perspective on advantages and disadvantages of blended cognitive behaviour therapy for depression: a qualitative content analysis. Psychother Res.

[CR24] van der Vaart R, Witting M, Riper H, Kooistra L, Bohlmeijer ET, van Gemert-Pijnen LJ (2014). Blending online therapy into regular face-to-face therapy for depression: content, ratio and preconditions according to patients and therapists using a Delphi study. BMC Psychiatry.

[CR25] Wentzel J, van der Vaart R, Bohlmeijer ET, van Gemert-Pijnen JE (2016). Mixing online and face-to-face therapy: how to benefit from blended care in mental health care. JMIR Ment Health.

[CR26] Wright JH, Wright AS, Albano AM, Basco MR, Goldsmith LJ, Raffield T, Otto MW (2005). Computer-assisted cognitive therapy for depression: maintaining efficacy while reducing therapist time. Am J Psychiatry.

[CR27] Zwerenz R, Baumgarten C, Becker J, Tibubos A, Siepmann M, Knickenberg RJ, Beutel ME (2019). Improving the course of depressive symptoms after inpatient psychotherapy using adjunct web-based self-help: follow-up results of a randomized controlled trial. J Med Internet Res.

[CR28] Zwerenz R, Becker J, Knickenberg RJ, Siepmann M, Hagen K, Beutel ME (2017). Online self-help as an add-on to inpatient psychotherapy: efficacy of a new blended treatment approach. Psychother Psychosom.

